# Comparison Between the Effects of Continuous and Intermittent Light-Intensity Aerobic Dance Exercise on Mood and Executive Functions in Older Adults

**DOI:** 10.3389/fnagi.2021.723243

**Published:** 2021-10-06

**Authors:** Kazuki Hyodo, Kazuya Suwabe, Daisuke Yamaguchi, Hideaki Soya, Takashi Arao

**Affiliations:** ^1^Physical Fitness Research Institute, Meiji Yasuda Life Foundation of Health and Welfare, Tokyo, Japan; ^2^Faculty of Health and Sport Sciences, Ryutsu Keizai University, Ibaraki, Japan; ^3^Sport Neuroscience Division, Advanced Research Initiative for Human High Performance (ARIHHP), Faculty of Health and Sport Sciences, University of Tsukuba, Ibaraki, Japan; ^4^Laboratory of Exercise Biochemistry and Neuroendocrinology, Faculty of Health and Sport Sciences, University of Tsukuba, Ibaraki, Japan

**Keywords:** aging, aerobic dance exercise, low intensity, interval exercise, exercise enjoyment, affective response, emotion, Stroop

## Abstract

There is a growing body of evidence suggesting that one bout of moderate-intensity exercise enhances executive functions in older adults. However, in terms of safety, feasibility, and continuity, older individuals prefer light, easy, and fun exercises to moderate and stressful exercises for improving executive functions. Therefore, light-intensity aerobic dance exercise (LADE) could be suitable if it produces potential benefits related to executive functions. As for continuous vs. intermittent exercise, intermittent exercise has received a lot of attention, as it results in greater effects on mood and executive functions than continuous exercise; however, its effects in older adults remain uncertain. Thus, in this study, we aimed to examine the acute effects of intermittent LADE (I-LADE) in comparison with those of continuous LADE (C-LADE) on mood and executive functions. Fifteen healthy older adults participated in 10-min I-LADE and C-LADE conditions on separate days. Perceived enjoyment following exercise was assessed using the Physical Activity Enjoyment Scale (PACES). The pleasantness of the mood during exercise and pleasure and arousal levels after exercise were assessed using the Feeling Scale and Two-Dimensional Mood Scale, respectively. Executive function was assessed using the Stroop task before and after exercise. As a result, pleasantness of the mood during exercise and exercise enjoyment levels were greater in I-LADE than in C-LADE. Arousal and pleasure levels and Stroop task performance increased after both LADEs and did not differ between the two exercise conditions. These findings suggest that although enhancement of mood and executive functions after exercise did not differ between C-LADE and I-LADE, I-LADE could be more enjoyable and fun than C-LADE. This study will help in the development of exercise conditions that can enable the elderly to enhance their executive functions in a fun way.

## Introduction

Previous studies have reported on the beneficial effects of physical activity or exercise in preventing age-related cognitive decline (Kramer et al., 1999; Hillman et al., 2008). In addition to the beneficial effect of chronic exercise (long-term regular exercise), several studies have reported that acute exercise, defined as a single bout of exercise, produces a positive effect on cognitive function in older adults. Many of these studies have focused on the effects of exercise on executive functions, a higher-order cognitive process compassing working memory, inhibitory control, planning to control goal-directed behavior (Miyake et al., 2000), and found that moderate-to-high intensity exercises, such as running and cycling (Kamijo et al., 2009; Hyodo et al., 2012; Johnson et al., 2016; Chang et al., 2019), resistance exercise (Johnson et al., 2016), and a combination of these exercises (Nouchi et al., 2020) enhanced these executive functions. However, it is difficult for older adults, who are anxious about their physical functions or whose level of motivation to exercise is low, to perform moderate-to-high intensity exercise and to continue regularly (Schutzer and Graves, 2004). Here, mood change during and after exercise is an important factor from the perspective of exercise motivation and effects on executive function. This is because exercise-related mood changes (positive affective response during exercise and exercise enjoyment) lead to the next exercise (Rhodes and Kates, 2015), and that exercise-induced mood changes (e.g., arousal or pleasure level) are associated with improved cognitive function (Byun et al., 2014; Nouchi et al., 2020; Suwabe et al., 2021). Therefore, it is necessary to clarify the most suitable characteristics of an exercise that is easy and fun for older adults to perform and that is effective for mood and executive functions.

In terms of the safety, feasibility, and enjoyment levels associated with an exercise for older adults, exercise characteristics, such as intensity, type, and mode, are important factors that should be examined carefully. Regarding the intensity of an exercise, light-intensity exercise is less stressful and easier for older adults to perform, which makes it safe and feasible to practice. As for the effect on mood, light-intensity exercise could induce a more pleasant mood during exercise than moderate-intensity exercise (Ekkekakis et al., 2011). With respect to the effect on executive functions, it is still under debate whether acute light-intensity exercise is effective for improving executive functions in older adults. For example, our previous study reported that 10 min of light-intensity cycling exercise enhanced executive functions in young adults, as assessed by the Stroop task (Byun et al., 2014). Dawe and Moore-Orr (1995), and Stones and Dawe (1993) reported that 15 min of light-intensity physical exercise (e.g., walking, whole body slow rhythmic movement while sitting) enhanced word fluency performance in older adults. In addition to aerobic exercise, recent studies have revealed the beneficial effect of yoga and virtual-reality training on executive functions (Wu et al., 2019; Burin et al., 2020; Burin and Kawashima, 2021). In contrast, Kamijo et al. (2009) reported that acute light-intensity cycling exercise did not improve executive functions assessed using the Flanker task.

As a type of exercise, dance appears to be suitable for older adults because of the familiarity with the older adults and the beneficial effect on mood and cognitive function. Based on data from the National Health and Nutrition Examination Surveys (NHANES), Fan et al. (2013) reported that the participation rate in dance as a leisure time physical activity was ranked second following walking among older American women. Moreover, as dance is cognitively and physically demanding (Brown et al., 2006), it is attracting attention as a type of exercise to enhance cognitive function effectively (Hewston et al., 2021). Kimura and Hozumi (2012) reported that acute light-intensity (40% ${\text{V˙o}}_{\text{2peak}}$) aerobic dance exercise (LADE) could enhance spatial working memory performance in older adults. Moreover, our recent study revealed that acute LADE improved vitality and pleasure levels as well as executive functions as evaluated by Stroop task performance (Hyodo et al., 2019). Moreover, in a systematic review, Predovan et al. (2019) reported that as an intervention, dance could help improve or maintain cognition in the elderly. Regarding the effect on mood, previous studies have revealed that participation in an ADE class instantly improves mood by, for example, increasing vigor and decreasing anger and depression (Maroulakis and Zervas, 1993; McInman and Berger, 1993; Pierce and Pate, 1994; Lane et al., 2003).

Whether an exercise is practiced continuously or intermittently could be another important characteristic that influences its effects. Continuous aerobic exercise (exercise in one continuous bout) has been widely reported to have positive effects on mood and executive functions in young and older adults (Yanagisawa et al., 2010; Hyodo et al., 2012; Hogan et al., 2013). In recent years, intermittent exercise (exercise in multiple short bouts with interval) has attracted a lot of attention for its time-efficient nature and beneficial effects not only on physical functions (Gibala et al., 2012; Tjønna et al., 2013) but also on mood and cognitive functions in young adults (Bartlett et al., 2011; Tsukamoto et al., 2016; Kujach et al., 2018; Ai et al., 2021). Several studies revealed intermittent exercise had a more positive effect on enjoyment and executive functions than continuous exercise probably *via* increased sense of achievement (Hoekstra et al., 2017), neural efficiency (Kao et al., 2017), or prefrontal activation (Lambrick et al., 2016). Most of the aforementioned studies, however, used intermittent high–intensity exercise, and to the best of our knowledge, no study has examined the effects of intermittent light-intensity exercise on mood and cognitive functions in older adults.

Based on the available evidence, we hypothesized that intermittent LADE (I-LADE) would be safe and fun for older adults to practice and would also be effective for maintaining and improving their mood and cognitive function. As the first step in examining this hypothesis, we aimed to examine the acute effects of I-LADE in comparison with those of continuous LADE (C-LADE) on mood and executive functions in older adults.

## Materials and Methods

### Participants

Fifteen older adults (aged 65–74 years) were recruited through advertisements in local magazines. The inclusion criteria for participation in this study were right-handedness; normal or corrected-to-normal vision; no history of any neuropsychiatric, neurological, or cardiovascular diseases; healthy cognitive abilities [Mini-Mental State Examination (MMSE) score > 28]; and the absence of depression [Geriatric Depression Scale (GDS) score <7]. All participants were physically active (exercising (e.g., walking, swimming) at least 2 days a week). Baseline characteristics are presented in Table 1.

**Table 1:** Demographic characteristics (*n* = 15).

	Male (*n* = 7)		Female (*n* = 8)	
	*M*	*SD*	*M*	*SD*
Age [years]	71.1	2.9	70	2.5
Height [cm]	164	4.8	150	4.4
Weight [kg]	60.3	5.6	47.8	6.6
BMI	22.4	1.4	21.2	2.0
Education [years]	13	2.5	13	1.9
MMSE [score]	29.3	0.95	28.6	1.2
GDS [score]	1.7	1.6	1.8	1.9

*M, mean; SD; standard deviation; BMI, body mass index; MMSE, Mini-Mental State Exam; GDS, Geriatric Depression Scale*.

Written informed consent was obtained from all the participants prior to the study. This study was approved by the Ethics Committee of the Physical Fitness Research Institute of Meiji Yasuda Life Foundation of Health and Welfare (approval number: 28003).

### Experimental Procedure

Each participant visited the laboratory three times on separate days. During the first visit, we checked their medical, cognitive, and psychological status using the MMSE and GDS questionnaires. Subsequently, they practiced the Stroop task, a psychological test for measuring executive functions, to familiarize themselves with the Stroop task, which was later used to assess executive functions; the participants performed the task twice. They also practiced an aerobic dance exercise program called “Slow Aerobic Dance Exercise” once.

**Figure 1:**
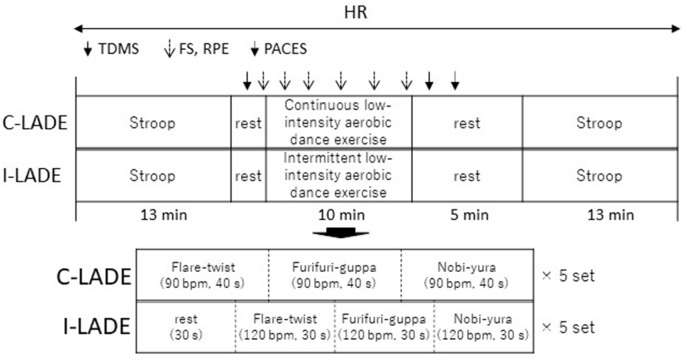
Experimental procedure. HR, heart rate; TDMS, Two-Dimensional Mood Scale; FS, Feeling Scale; RPE, rating of perceived exertion; PACES, Physical Activity Enjoyment Scale.

During the second and third visits, we assessed the acute effects of C-LADE or I-LADE on mood and executive functions using a counterbalanced, within-subjects design. The two exercise conditions were separated by an average of 5 days (2–12 days). The experimental procedure is depicted in Figure 1. First, the participants performed the Stroop task (pre-session) following a short practice (20 trials). Next, their mood was assessed using the Two-Dimensional Mood Scale (TDMS). Subsequently, they performed either I-LADE or C-LADE for 10 min. The Feeling Scale (FS) score and ratings of perceived exertion (RPE) were obtained verbally from each participant before the exercise and at 2, 4, 6, 8, and 10 min after beginning the exercise. Heart rate (HR) was monitored throughout the experimental period using Polar H7 (Polar Electro Oy, Kempele, Finland). Immediately after the exercise, the participant completed the TDMS and the Physical Activity Enjoyment Scale (PACES) questionnaires. Finally, they performed the Stroop task (post-session) 5 min after the end of the exercise. The order of the exercise conditions was counterbalanced across participants.

### Exercise Intervention

**Figure 2:**
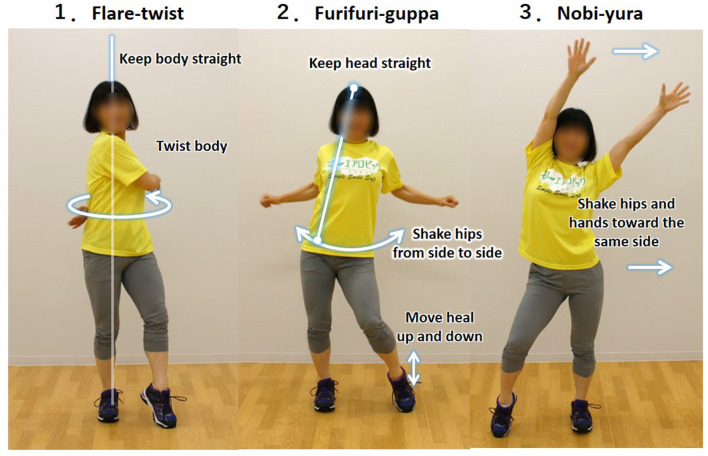
Three components of the “Slow Aerobic Dance Exercise”.

We used the “Slow Aerobic Dance Exercise” as the LADE intervention in this study (Figure 2). The intervention mainly comprised three dynamic upper-body stretches: (1) twisting the upper body; (2) pulling the elbows back and then clapping the hands while shaking the waist from side to side; and (3) waving the arms like wiping the windows while shaking the waist from side to side. The C-LADE condition comprised of five sets of the three movements, each of which was routinely executed for 40 s. The exercise tempo was set to 90 bpm. In contrast, the I-LADE condition comprised of five sets of the three movements for 90 s with rest intervals of 30 s. The exercise tempo was set to 120 bpm. The number of times each movement was repeated was equal between the two LADE conditions. Each participant conducted each LADE movement alone while watching a tutorial video with the original music (Witek et al., 2014).

### Exercise Intensity

To verify that the exercises were in light-intensity range, we evaluated objective and subjective exercise intensity using HRR and RPE, respectively. We calculated the heart rate reserve (HRR) for each participant using the following equation:


$$\text{HRR}=\frac{\text{HR during exercise}-\text{resting HR}}{\text{predicted max HR}-\text{resting HR}}*100\;(\%)$$

The age-predicted maximum HR was calculated using the following equation (Tanaka et al., 2001):


208−0.7×age

For subjective exercise intensity, the mean RPE during exercise was used.

### Mood-Related Variables

To assess exercise enjoyment levels, we used the Japanese version of the PACES (Kendzierski and DeCarlo, 1991; Marcus and Forsyth, 2006). The PACES consists of 18 items scored on a 7-point bipolar scale. The minimum total score is 18, and the maximum total score is 126.

To assess the participant’s mood during the exercise, we used the FS comprising of a single item (Hardy and Rejeski, 1989). The item assesses how the participant currently feels (pleasant–unpleasant) about the exercise on an 11-point bipolar scale with scores ranging from -5 (very bad) to + 5 (very good). We calculated the mean FS score during exercise to assess whether the mood was pleasant while exercising.

We used different questionnaires to evaluate the participants’ mood during and after exercise to minimize common-method variance. To assess the mood after the exercise, we used the TDMS (Sakairi et al., 2013), which comprise eight mood-expressing words related to both pleasure and arousal states (i.e., energetic, lively, lethargic, listless, relaxed, calm, irritated, and nervous). Participants scored each item using a 6-point rating scale ranging from 0 (not at all) to 5 (extremely). Pleasure and arousal levels (ranging from -20 to + 20 points) were determined by calculations based on each score.

### Executive Functions

We assessed executive functions using a computer-based color-word matching Stroop task (Zysset et al., 2001; Hyodo et al., 2016). Two rows of letters were presented on a computer screen. The participants were required to determine whether the colors of the letters in the top row matched the color names printed in the bottom row and to press the corresponding “yes” or “no” button as quickly and accurately as possible with their forefingers. The task comprised 30 neutral and 30 incongruent trials presented in a random order. For neutral trials, the letter sequence “XXXX” was displayed in the top row in red, green, blue, or yellow, and the word “
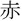
” (red in English), “
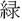
” (green in English), “
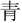
” (blue in English), or “
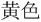
” (yellow in English) was displayed in the bottom row in black. For incongruent trials, the top row contained the word “
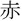
” “
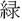
” “
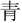
” or “
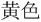
” displayed in an incongruent color (e.g., “
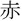
” in green). All the word stimuli were displayed in Japanese. To achieve sequential visual attention, the top row appeared 350 ms earlier than the bottom row. Each stimulus remained on the screen for 3 s. The protocol contained an equal number of trials with “yes” as the correct answer and “no” as the correct answer. Between trials, fixation cross was presented for 9–12 s (inter stimulus interval) to avoid the prediction of the timing in the subsequent trial. The accuracy and correct reaction time were measured. Stroop interference time (i.e., the difference in the correct reaction time between incongruent and neutral trials) was used as an index of executive functions, similar to in previous studies (Yanagisawa et al., 2010; Hyodo et al., 2012; Byun et al., 2014; Ochi et al., 2018).

### Statistical Analyses

The HRR, RPE, and PACES scores were compared between the two exercise conditions using a paired t-test. For the FS scores, TDMS scores, and Stroop interference times, a two-way repeated measures analysis of variance (ANOVA) including exercise condition (C-LADE/I-LADE) and time (pre/post [or during] exercise) was conducted. If a significant interaction was observed, changes in the values (post-pre) were compared between the exercise conditions using an unpaired *t*-test. In addition, for the variables with significant differences between exercise conditions, Pearson correlation analysis was performed to investigate the relationship between them. All statistical analyses were conducted using R version 3.5.1 (R Core Team, 2018). Statistical significance was set at *p* < 0.05 for all the analyses.

## Results

### Exercise Intensity

The average HRR during C-LADE and I-LADE was 19.4 ± 6.50 and 22.4 ± 6.91%, respectively. The average RPE during C-LADE and I-LADE was 10.3 ± 1.69 and 10.6 ± 1.9%, respectively. There were no significant differences in the mean HRR (*t*_(14)_ = 1.77, *p* = 0.10, *d* = 0.46) or RPE (*t*_(14)_ = 1.01, *p* = 0.31, *d* = 0.28) between the two exercise conditions.

### Mood-Related Variables

**Figure 3:**
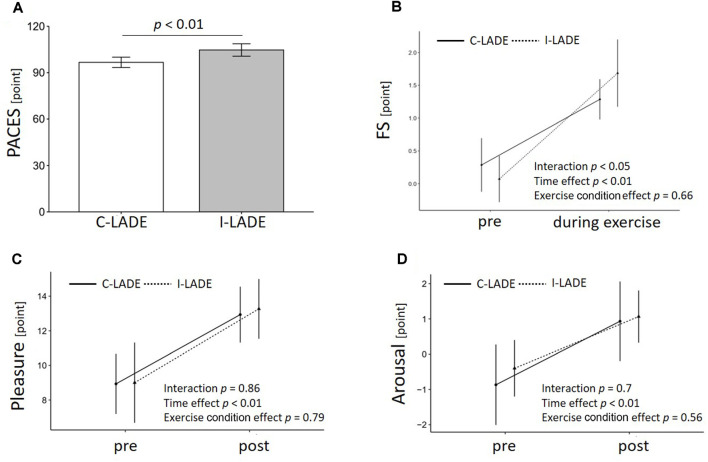
Results of mood-related variable analysis. Means and standard deviations are shown. The *p* values in **(A)** show the results of the paired *t*-test. The *p* values in **(B)**, **(C)**, and **(D)** show the results of the two-way ANOVA with time and exercise condition as factors. PACES, Physical Activity Enjoyment Scale; FS, Feeling Scale; ANOVA, analysis of variance.

Figure 3 presents the PACES, FS, and TDMS scores. Data of the one participant who had the maximum FS score (5 points) before exercise were excluded from the FS score analysis. The PACES score was significantly higher after I-LADE than after C-LADE (*t*_(14)_ = 3.10, *p* < 0.01, *d* = 0.80; Figure 3A).

The two-way ANOVA for the FS score showed a significant interaction (*F*_(1, 13)_ = 6.24, *p* = 0.023, $\eta_{\text{p}}^{2}$ = 0.324) and main effect of time (*F*_(1, 13)_ = 35.24, *p* < 0.01, $\eta_{\text{p}}^{2}$ = 0.732) but no significant main effect of the exercise condition (*F*_(1, 13)_ = 0.21, *p* = 0.66, $\eta_{\text{p}}^{2}$ = 0.016). The change in the FS score was significantly higher in I-LADE than in C-LADE (*t*_(13)_ = 2.31, *p* = 0.049, *d* = 0.80; Figure 3B).

Regarding the TDMS results, the two-way ANOVA for the pleasure level showed a significant main effect of time (*F*_(1, 14)_ = 14.42, *p* < 0.01, $\eta_{\text{p}}^{2}$ = 0.507). However, there was no significant interaction (*F*_(1, 14)_ = 0.03, *p* = 0.86, $\eta_{\text{p}}^{2}$ = 0.002) or main effect of the exercise condition (*F*_(1, 14)_ = 0.08, *p* = 0.79, $\eta_{\text{p}}^{2}$ = 0.005). In addition to the pleasure level, the arousal level showed a significant main effect of time (*F*_(1, 14)_ = 15.05, *p* < 0.01, $\eta_{\text{p}}^{2}$ = 0.518) but no significant interaction (*F*_(1, 14)_ = 0.15, *p* = 0.7, $\eta_{\text{p}}^{2}$ = 0.011) or main effect of the exercise condition (*F*_(1, 14)_ = 0.35, *p* = 0.56, $\eta_{\text{p}}^{2}$ = 0.024).

### Executive Functions

**Figure 4:**
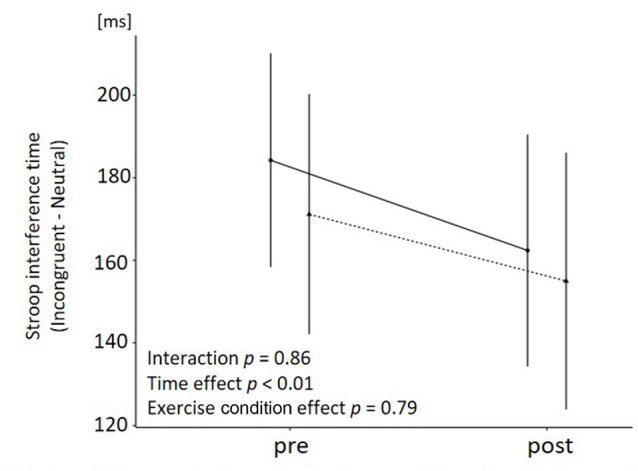
Change in Stroop interference time for each condition. Mean and standard deviation are shown. The *p* value shows the results of the two-way ANOVA with time and exercise condition as factors.

Table 2 presents the accuracy rates and reaction times (RT) in the neutral and incongruent trials before and after the exercises. To examine the acute effect of the exercise on executive functions, Stroop interference time (RT difference between incongruent and neutral trials) was analyzed. The two-way ANOVA revealed a significant main effect of time in the case of Stroop interference time (*F*_(1, 14)_ = 4.74, *p* = 0.05, $\eta_{\text{p}}^{2}$ = 0.253), which showed that Stroop interference times were significantly shorter during the post-session than during the pre-session (Figure 4). However, we found no significant interactions (*F*_(1, 14)_ = 0.03, *p* = 0.86, $\eta_{\text{p}}^{2}$ = 0.002) or main effects of the exercise condition (*F*_(1, 14)_ = 0.47, *p* = 0.50, $\eta_{\text{p}}^{2}$ = 0.033).

**Table 2:** Behavioral data based on the Strooptask.

		C-LADE				I-LADE			
*n* = 15		Pre-session		Post-session		Pre-session		Post-session	
		*M*	*SD*	*M*	*SD*	*M*	*SD*	*M*	*SD*
AC [%]
	Neutral	99.3	1.9	99.8	0.9	99.3	1.4	99.1	1.5
	Incongruent	96.7	3.6	96.4	3.7	98.0	3.0	96.9	3.9
RT [ms]
	Neutral	744.6	130.6	729.4	96.7	756.3	129.9	716.0	112.4
	Incongruent	928.9	219.4	891.8	202.8	927.4	231.2	870.9	177.4

*I-LADE, intermittent light-intensity aerobic dance exercise; C-LADE, continuous light-intensity aerobic dance exercise; AC, accuracy; RT, reaction time; M, mean; SD, standard deviation*.

### Correlations Between Significant Variables

We found significant differences in the PACES and FS scores between exercise conditions, Pearson’s correlation analysis was conducted to assess the relationship between the difference in FS score changes (ΔFS in I-LADE − ΔFS in C-LADE) and the difference in PACES score [PACES in I-LADE − PACES in C-LADE]. There was no significant correlation between them (*r* = 0.097, *p* = 0.74). This result suggests that different factors are involved in the differences in FS and PACES between exercises.

## Discussion

In this study, we aimed to clarify the acute effects of I-LADE on mood and executive function compared with those of C-LADE. We hypothesized that I-LADE would induce greater acute effects of the exercise on psychological arousal and pleasure levels, enjoyment levels, and executive functions compared with C-LADE. This hypothesis was partially confirmed in this study. The results of this study showed that the participants experienced higher pleasure levels during I-LADE than during C-LADE and greater exercise enjoyment in I-LADE than in C-LADE. However, the changes in arousal, pleasure and executive function before and after the exercise did not differ between the exercise conditions. These findings suggest that I-LADE is more fun to perform than C-LADE, but the enhancing effects of the exercise on mood and executive function after exercise are not different between the exercise conditions in older adults.

Regarding the exercise intensity, although it was not significant, a low effect size was observed between exercise conditions. In this regard, HRR in both exercises were within the range of light-intensity, and the difference in the mean of HRR between the two exercises (3%) is considered to be a small difference with little effect on physiological responses (metabolism, respiratory system, etc.; Sanada et al., 2007) and affective responses (Ekkekakis et al., 2004). Moreover, the difference in HRR between the exercises was not correlated with the differences in FS and PACES (*r* = -0.12, *r* = -0.18) between the exercises. These results suggest that the different responses in FS and PACES between the exercises could be due to the different exercise modes instead of possible small intensity differences.

The result of the exercise enjoyment in this study was consistent with those of previous studies reporting that intermittent exercise was more enjoyable than continuous exercise in young adults (Bartlett et al., 2011; Martinez et al., 2015; Hoekstra et al., 2017). This result suggests that intermittent exercise may be more enjoyable than continuous exercise in older adults, even at light-intensity. Exercise enjoyment has been related to cognitive appraisal, such as mastery of an exercise or a sense of achievement (Kilpatrick et al., 2015; Hoekstra et al., 2017). Consequently, it is possible that the I-LADE condition involving a larger number of shorter sessions enhanced the sense of accomplishment compared with the C-LADE condition involving only one session, which may have led to more enjoyment.

As for the greater effect on FS during I-LADE, this result would be inconsistent with previous studies using intermittent high–intensity exercise. A meta-analysis examining the difference in the effect on mood between interval exercise and continuous exercise reported that the mood during interval high-intensity exercise is less pleasant than that during continuous moderate-intensity exercise; however, there was no difference in mood when the intensity was high in both cases (Niven et al., 2020). The difference in the results between this study and previous studies could be explained by the dual-mode theory (DMT; Ekkekakis, 2003). According to this theory, the mood during exercise is controlled by the interaction between cognitive parameters, such as self-efficacy and interoceptive cues, such as ventilation and acidosis. For instance, when exercise intensity is below the ventilatory threshold or lactate threshold, cognitive parameters positively influence the mood. However, when exercise intensity is above these thresholds, interoceptive cues negatively influence the mood. Based on the DMT, since exercise intensity could strongly influence the mood during exercise at above moderate intensities, the influence of continuous or intermittent exercise on the mood during exercise could be minimal. In contrast, since both I-LADE and C-LADE in this study were light-intensity conditions, the differences in exercise characteristics (intermittent or continuous) could influence the mood. I-LADE might have a greater effect on self-efficacy or self-esteem during exercise than C-LADE, which could lead to a more pleasant mood during exercise.

Although the mood during I-LADE was more pleasant than that during C-LADE, the pleasure level measured by the TDMS immediately after the exercise was not different between the two exercise conditions. These results indicated that the more pleasant mood during I-LADE than during C-LADE was not maintained after the exercise. This result of no difference in mood after exercise is consistent with previous studies reporting that exercise intensity affected the mood during exercise, but its effect disappeared after exercise (Kilpatrick et al., 2007; Ekkekakis et al., 2011). As the explanation of this result, the previous studies pointed out that the affective response during exercise would be strongly driven by core response directly by bodily sensations, but it disappeared after the exercise (Kilpatrick et al., 2007; Martinez et al., 2015). Applying this explanation to the present study, it is possible that during the exercise, the pleasure was increased in response to more bodily sensations of rhythmic and varied movements in I-LADE compared to C-LADE. However, after the exercise, the stimulation of body movement disappeared, which may lead to the lack of difference between exercise conditions. Although the effect of the exercise mode on the mood might disappear immediately after exercise, a previous study reported that mood changes during exercise are related to future physical activity (Rhodes and Kates, 2015). Therefore, a more pleasant mood during I-LADE might be meaningful in terms of promoting physical activity or exercise in the future.

Regarding the psychological arousal levels, although both LADEs increased the arousal levels, there was no significant difference between these levels. Exercise intensity is strongly associated with psychological and physiological arousal levels (Ekkekakis, 2003). In this study, since there was no significant difference in exercise intensity between the two conditions, no significant difference in the arousal response to the exercise seemed to be observed between them.

As for the executive function, the Stroop interference time shortened after both exercise conditions, and no significant difference in the pattern of change was observed between them. These results suggest that the difference in the exercise mode (intermittent or continuous) does not produce the difference in the exercise-induced changes in executive functions among older adults when the exercise is conditioned for light-intensity and aerobic dance. This result is inconsistent with previous studies that intermittent exercise improved executive task performance such as Stroop task (Lambrick et al., 2016; Tsukamoto et al., 2016) and Flanker task (Kao et al., 2017) compared to continuous exercise. These studies compared high-intensity intermittent exercise and moderate-intensity continuous exercise, but no study has compared the effects within the same range of exercise intensity. Then, within the same range of exercise intensity, the effects of intermittent and continuous exercise on executive functions may not be different. Although the exercise mode and intensity are different from our study, this assumption was supported by a previous study reporting that both aerobic and resistance exercises with moderate intensity improved Stroop task performance to the same degree in older adults (Johnson et al., 2016). In addition, previous studies (Byun et al., 2014; Nouchi et al., 2020; Suwabe et al., 2021) reported that the improved executive function was associated with improved mood (e.g., arousal and vitality). These findings suggest that the lack of LADE-induced differences in the Stroop interference time change in this study might be due to the lack of differences in the mood change such as pleasure and arousal between the two exercises. Lastly, since the exercise intensity of the aerobic dance exercise in this study was light, the improvement effect on executive functions may have been small (Kamijo et al., 2009), resulting in no difference. That is, there was no control condition in this study, we cannot deny the possibility that the shortening in both conditions was due to a learning/habituation effect. In this regard, however, we believe that there was little impact on the learning effect from the following two points: (1) participants became familiar with the Stroop task during the practice day, and there was no difference in Stroop interference time between the first and second practice sessions (Supplementary Table 1); and (2) the results of previous studies in the same condition repeatedly showed that the resting control did not change Stroop interference time (Yanagisawa et al., 2010; Hyodo et al., 2012; Byun et al., 2014; Kujach et al., 2018). To compare the effects of intermittent and continuous aerobic dance exercise on executive functions in more detail, it is necessary to compare the effects of intermittent and continuous exercise with a control condition and at various intensities in the future.

This study has some limitations. First, as mentioned above, there was no resting control condition. The improvement of mood and executive function might not only be due to the exercise effect. Second, because of the small sample size in this study, statistical power might be too low to detect the difference in the effects between the two exercise conditions. This means that a clear conclusion should be avoided for the variables showing no significant difference between the two exercise conditions. Third, the participants in this study were relatively physically active, it is unclear whether the results apply to inactive older adults. Lastly, as we used only the Stroop task to assess executive functions, it is still unknown whether other aspects of executive functions, such as cognitive flexibility and updating, are differently affected by the difference in the mode of dance exercise program.

In conclusion, although performing LADE continuously or intermittently may not affect the enhancement of mood and executive function after exercise in older adults, intermittent exercise could be more enjoyable and fun than continuous exercise. Consequently, intermittent exercise could have the potential to improve mood and executive functions, as well as to promote a higher adherence level to exercise regimens in older adults, even in the case of light-intensity exercises. This study may help to develop exercise programs that can be performed by many older adults to enhance their mood and executive functions. Further studies with a larger sample including inactive people are needed to further clarify the effect of the exercise.

## Data Availability Statement

The raw data supporting the conclusions of this article will be made available by the authors, without undue reservation.

## Ethics Statement

The studies involving human participants were reviewed and approved by Ethics Committee of the Physical Fitness Research Institute of Meiji Yasuda Life Foundation of Health and Welfare (approval number: 28003). The patients/participants provided their written informed consent to participate in this study.

## Author Contributions

KH contributed to the conceptualization, data correction, data analysis, and writing of the first draft of this manuscript. KS contributed to the conceptualization, methodological development, data analysis, and manuscript revision. DY contributed to data correction and manuscript revision. HS contributed to project funding and manuscript revision. TA contributed to the data analysis and revision of the manuscript. All authors contributed to the article and approved the submitted version.

## Conflict of Interest

The authors declare that the research was conducted in the absence of any commercial or financial relationships that could be construed as a potential conflict of interest.

## Publisher’s Note

All claims expressed in this article are solely those of the authors and do not necessarily represent those of their affiliated organizations, or those of the publisher, the editors and the reviewers. Any product that may be evaluated in this article, or claim that may be made by its manufacturer, is not guaranteed or endorsed by the publisher.
