# Supplementary material for: Comparison Between the Effects of Continuous and Intermittent Light-Intensity Aerobic Dance Exercise on Mood and Executive Functions in Older Adults
Source: Front Aging Neurosci. 2021 Oct 6;13:723243. doi: 10.3389/fnagi.2021.723243 (PMC8577647; doi:10.3389/fnagi.2021.723243)
Supplement: Supplementary file 1 [file Table_1.pdf]

Supplementary Table 1. Stroop task performance in practice session

n = 15		Practice				t	*p*-value	Cohen's d
		first		second				
		M	*SD*	*M*	*SD*			
AC [%]								
	Neutral	99.8	0.9	98.7	1.7	-2.65	0.02	-0.68
	Incongruent	96.2	4.7	95.8	3.4	-0.27	0.79	-0.07
RT [msec]								
	Neutral	838.2	164.2	805.9	153.0	-1.09	0.29	-0.28
	Incongruent	1037.1	185.2	1028.5	226.2	-0.33	0.75	-0.09
	Interference	198.9	109.7	222.5	117.0	0.73	0.47	0.19

AC; Accuracy, RT; reaction time, SD; standard deviation
